# Parathyroid hormone therapy improves MRSA-infected fracture healing in a murine diabetic model

**DOI:** 10.3389/fcimb.2023.1230568

**Published:** 2023-09-27

**Authors:** Hyuk-Kwon Kwon, Sean V. Cahill, Kristin E. Yu, Kareme D. Alder, Christopher M. Dussik, Jain Jeong, Jung Ho Back, Francis Y. Lee

**Affiliations:** ^1^ Department of Orthopaedics and Rehabilitation, Yale University School of Medicine, New Haven, CT, United States; ^2^ Division of Life Science, Gyeongsang National University, Jinju, Republic of Korea; ^3^ Department of Internal Medicine, Section of Digestive Diseases, Yale School of Medicine, New Haven, CT, United States

**Keywords:** diabetes, MRSA, fracture, nonunion, osteomyelitis, parathyroid hormone

## Abstract

**Introduction:**

Diabetes mellitus (DM) impairs fracture healing and is associated with susceptibility to infection, which further inhibits fracture healing. While intermittent parathyroid hormone (1-34) (iPTH) effectively improves fracture healing, it is unknown whether infection-associated impaired fracture healing can be rescued with PTH (teriparatide).

**Methods:**

A chronic diet-induced type 2 diabetic mouse model was used to yield mice with decreased glucose tolerance and increased blood glucose levels compared to lean-fed controls. Methicillin-resistant *Staphylococcus aureus* (MRSA) was inoculated in a surgical tibia fracture model to simulate infected fracture, after which mice were treated with a combination of antibiotics and adjunctive teriparatide treatment. Fracture healing was assessed by Radiographic Union Scale in Tibial Fractures (RUST), micro-computed tomography (μCT), biomechanical testing, and histology.

**Results:**

RUST score was significantly poorer in diabetic mice compared to their lean nondiabetic counterparts. There were concomitant reductions in micro-computed tomography (μCT) parameters of callus architecture including bone volume/total volume, trabecular thickness, and total mineral density in type 2 diabetes mellitus (T2DM) mice. Biomechanicaltesting of fractured femora demonstrated diminished torsional rigidity, stiffness, and toughness to max torque. Adjuvant teriparatide treatment with systemic antibiotic therapy improved numerous parameters of bone microarchitecture bone volume, increased connectivity density, and increased trabecular number in both the lean and T2DM group. Despite the observation that poor fracture healing in T2DM mice was further impaired by MRSA infection, adjuvant iPTH treatment significantly improved fracture healing compared to antibiotic treatment alone in infected T2DM fractures.

**Discussion:**

Our results suggest that teriparatide may constitute a viable adjuvant therapeutic agent to improve bony union and bone microarchitecture to prevent the development of septic nonunion under diabetic conditions.

## Introduction

Following fracture, bone has the unique potential to regenerate with the same material and structural properties of the original tissue. Normal fracture healing proceeds without scarring and residual functional deficit (Giannoudis et al., 2007; Einhorn and Gerstenfeld, 2015). However, it has been estimated that about 100,000 fractures in the United States fail to adequately heal annually, resulting in nonunion (Hak et al., 2014). The overall nonunion rate among diabetic and nondiabetic individuals is estimated at 5%–10% (Beaver et al., 1997; Antonova et al., 2013). Delayed fracture union and nonunion contribute to the burden of pain, disability, and reduced quality of life (QoL) endured by patients, with reduced QoL levels comparable to those of congestive heart failure, stroke, and acquired immunodeficiency syndrome (Brinker et al., 2013; Schottel et al., 2015; Brinker et al., 2017).

A major risk factor for fracture nonunion is diabetes mellitus (DM), a multisystem metabolic and inflammatory condition characterized by hyperglycemia and altered insulin signaling. DM may be characterized as type 1—autoimmune destruction of beta-islet pancreatic cells that produce insulin—or type 2, which is associated with tissue insulin resistance, central obesity, dysregulated adiposity, and eventual insulin production deficits. In addition, 90%–95% of cases of DM may be attributed to type 2 diabetes mellitus (T2DM) (Henderson et al., 2019). DM impairs fracture healing and is associated with higher rates of delayed union and nonunion. This perturbation of fracture healing may be related to the reduction of stability at the fracture site, interruption of bone metabolism, and induction of abnormal inflammatory responses in the setting of DM (Bouillon, 1991; Blakytny et al., 2011; Brown et al., 2014; Henderson et al., 2019). DM also predisposes patients to infection, which can be devastating to the diabetic bone fracture healing response (Carey et al., 2018). The most common causative agent of musculoskeletal infection is *Staphylococcus aureus*, and resistant strains such as methicillin-resistant *Staphylococcus aureus* (MRSA) pose an increased risk to recalcitrant osteomyelitis and septic arthritis (Ellington et al., 2006; Calhoun et al., 2009; Garzoni and Kelley, 2009; Lindsey et al., 2010; de Mesy Bentley et al., 2017; Johnson et al., 2018; Johnson et al., 2019; Yu et al., 2020a; Kwon et al., 2021; Kwon et al., 2022a; Kwon et al., 2022b; Yu et al., 2022; Kwon et al., 2023). Systemic antibiotics are a prerequisite for open fractures with highly suspicious bacterial contamination (Burke, 1961; Gillespie and Walenkamp, 2001; Penn-Barwell et al., 2012; Carver et al., 2017; Sweet et al., 2018).

Teriparatide is a peptide drug that includes the active component of parathyroid hormone [PTH (1-34)], which is proven to be the first Food and Drug Administration (FDA)-approved anabolic agent to treat osteoporosis. Teriparatide exerts an anabolic effect on bone by stimulating osteoblast differentiation and proliferation from precursor cells (Neer et al., 2001; Aspenberg and Johansson, 2010; Aspenberg et al., 2010; Canintika and Dilogo, 2020). While not a first-line agent in the treatment of osteoporosis, teriparatide has been demonstrated to reduce the risk of vertebral and non-vertebral fractures while increasing bone mineral density in postmenopausal women in multiple randomized controlled trials (Neer et al., 2001; Greenspan et al., 2007; Warden et al., 2009; Aspenberg and Johansson, 2010; Aspenberg et al., 2010; Alder et al., 2020b). Administration of intermittent systemic PTH was associated with significant improvement in the rate of fracture healing, partial reversal of defective bone mineralization, and trabecular bone deficits observed in animal models of T2DM (Aspenberg and Johansson, 2010; Aspenberg et al., 2010; Alder et al., 2020b).

While iPTH may improve fracture healing in DM animal models, it remains to be elucidated whether infection-associated nonunions in T2DM mice can be rescued by iPTH. The primary objective of this study was to determine whether the adjuvant iPTH with conventional antibiotic therapy improves open-infected fracture healing in both diabetic and normal mice.

## Materials and methods

### Type 2 diabetic mouse model

All animal experiments were approved by the Yale University Institutional Animal Care and Use Committee (IACUC; Number: 2020-20129). Male C57BL/6J mice aged 4 weeks were purchased from Jackson Laboratories (Bar Harbor, ME, USA). Animals were housed at 22°C ± 3°C on a 12-h light/dark cycle in ventilated cages and were given food and water *ad libitum*. Chronic high-fat and high-sugar (HFHS) diet (40 kcal% fat and 40 kcal% sucrose; Research Diets Inc., New Brunswick, USA; cat. number: D12327) for 6 months resulted in obese mice with decreased glucose tolerance and increased blood glucose levels compared to lean-fed controls (Farnsworth et al., 2018).

### Glucose tolerance analysis

All blood glucose measurements were obtained using a commercially available One-Touch^®^ Ultra^®^2 Glucometer (LifeScan, Milpitas, CA, USA) using blood collected from the tail vein. The maximum glucometer reading was 600 mg/dL. Mice underwent body weight measurement and glucose tolerance testing after 3 months on the lean and HFHS diet to establish a chronic diabetic model. Mice were fasted overnight before the glucose tolerance test (GTT). Baseline fasting blood glucose levels were measured. An intraperitoneal injection of 20% glucose solution (Teknova Inc., Hollister, CA, USA; cat. number: G0530) was given, with a total glucose load of 2 g/kg. Blood glucose levels were measured at 15, 30, 60, and 120-min intervals following injection. Geometric areas under the curves (AUCs) were calculated for comparison of glucose tolerance. Mice underwent body weight, fasting blood glucose, and glucose tolerance testing after 6 months on the normal and HFHS diet.

### Histological analysis of fatty liver

Liver tissues on normal and HFHS diet at 6 months were fixed with 10% formalin solution (Thermo Fisher Scientific, Inc., Waltham, MA, USA; cat. number: 23-245684) and then embedded with paraffin. Paraffin sections were stained with hematoxylin and eosin solution for histopathological analysis. All stained slides were scored for hepatic parenchymal involvement by steatosis, liver cell injury, and inflammation using a scoring system described by Kleiner et al. (2005). A total hepatic pathologic score was generated by adding the individual scores for steatosis, liver cell injury, and inflammation.

### MRSA inoculum preparation

The USA300-FPR3757 strain of MRSA expressive of green fluorescent protein (GFP) was provided by Dr. Alice Prince at Columbia University, New York, USA. MRSA was transferred onto Mueller–Hinton agar plates (Sigma-Aldrich Co., St. Louis, MO, USA; cat. number: 70191) containing oxacillin (6 µg/mL; Sigma-Aldrich Co.; cat. number: 1481000) and incubated in a 35°C incubator for 24 h. Single MRSA colonies were also planktonically cultured in lysogeny broth (LB; Invitrogen, Carlsbad, CA, USA; cat. number: 10855021) containing oxacillin (6 µg/mL; Sigma-Aldrich Co.) for 24 h (Kwon et al., 2023).

### Surgical open tibial fracture model

Animals underwent surgery at 7–8 months of age after induction of T2DM phenotypes. The tibial fracture surgical procedure was based on previous fracture healing studies by authors and other investigators (Alder et al., 2020b; Cahill et al., 2021; Hao et al., 2021). Anesthesia was achieved with ketamine (50 mg/kg) and xylazine (5 mg/kg). Depth was assessed by pedal withdrawal. Buprenorphine (0.1 mg/kg intraperitoneal) and bupivacaine (4 mg/kg subcutaneous) were given preemptively according to institutional animal care guidelines. The hair of the right leg was removed using Veet^®^ In Shower Cream (Reckitt Benckiser, Slough, England, UK), and the skin was disinfected with povidone-iodine and isopropyl alcohol pads (Professional Disposables International, Inc., NY, USA). An incision was made over the right tibia. The patella was lateralized, and a 25-gauge needle was used to predrill a hole in the cortical bone of the proximal tibia shaft. A scalpel was used to create a transverse, low-energy, non-comminuted, mid-shaft fracture just distal to the tibial prominence. The fracture was stabilized with a 0.35-mm insect pin inserted at the proximal tibia. Care was used to minimize bleeding and surrounding tissue damage. In this study, 5 μL of phosphate buffered saline (PBS) containing MRSA [1 × 10^6^ colony-forming units (CFU)] was applied to the fracture site. An intraoperative incubation period of 10 min was allowed to facilitate penetration of tissues prior to closure. The fracture site was dried of excess moisture with sterile gauze. The wound was closed using a simple interrupted technique with Coated Vicryl Suture (Ethicon Inc., Somerville, New Jersey, USA; cat. number: J391H) followed by sterile surgical staples using AutoClip^®^ System (Fine Science Tools USA, Inc., Foster City, CA, USA; cat. number: 12020-00). All mice were then placed on a warming pad and monitored until ambulatory.

### Antibiotic and teriparatide treatment

Mice in systemic antibiotic therapy groups were given subcutaneous injections of vancomycin (40 mg/kg; Sigma-Aldrich Co.; cat. number: 1709007) and rifampin (25 mg/kg; G-Biosciences, St. Louis, MO, USA; cat. number: RC-192). Antibiotic type and dosing were based on previous mouse studies investigating staphylococcal implant and bone infections, which demonstrated the efficacy of combinatory antibiotic therapy (Cahill et al., 2021). Mice were treated over a 3-day period, with one injection immediately following surgery and then 24 and 48 h later. For mice receiving systemic iPTH [human PTH (1-34); Alfa Aesar, Haverhill, MA, USA; cat. number: J66180] treatment, a subcutaneous injection of iPTH (40 µg/kg/day) was given immediately following surgery 5 days per week over 28 days for a total of 20 injections.

### Colony-forming unit analysis

Blood, fractured tibiae, and intramedullary pins were harvested. The implant device was transferred to 1 mL Dulbecco’s phosphate buffered saline (DPBS; Thermo Fisher Scientific, Inc.) solution and vortexed (Vortex Genie 2; Scientific Industries Inc., Bohemia, New York, USA) at 3,000 rpm for 10 min to detach MRSA from the bony implant. Samples [blood (100 µL), DPBS (100 µL), and tissue cells (1 × 10^6^)] were seeded on Mueller–Hinton agar plates (Sigma-Aldrich Co.) containing oxacillin (6 µg/mL; Sigma-Aldrich Co.) and incubated in a 35°C incubator for 24 h. CFU was detected with the ChemiDoc™ Touch Imaging System (Bio-Rad Laboratories, Hercules, CA, USA) and quantified using ImageJ software (National Institutes of Health, Bethesda, MD, USA) (Schneider et al., 2012).

### Radiographic and histologic fracture healing analysis

Anterior-posterior (AP) and lateral radiographs of tibiae sacrificed 28 days postoperatively were obtained by the author using *In-Vivo* Multispectral FX pro (Bruker, Madison, WI, USA). Healing was assessed by the Radiographic Union Scale in Tibial Fractures (RUST) score. A score of 10 or greater was considered radiographic union as previously validated (Cahill et al., 2021). Fractured tibiae were randomly assigned to histologic analysis, with additional histology samples harvested at 14 and 28 days. Histology tissues were fixed in 10% formalin solution (Thermo Fisher Scientific, Inc.) and decalcified in 10% EDTA solution (Sigma-Aldrich Co.) for 3 weeks at 4°C. Intramedullary pins were removed before histologic analysis. Tissues were dehydrated with graded alcohols, cleared with xylene, and paraffinized using the Tissue Tek VIP tissue processor (Sakura Finetek, Torrance, CA, USA). Tissues were cut into sections 5 µm thick, mounted onto slides, and stained with hematoxylin and eosin, Safranin O, and Gram stains. Histology images were obtained using BioTek Cytation™ 5 Cell Imaging Multi-Mode Reader and BioTek Gen5 software (BioTek Instruments Inc., Winooski, VT, USA).

### Micro-computed tomography analysis

Whole tibiae were used for micro-computed tomography (µCT) analysis of cancellous bone inside the callus with the Scanco µCT 50 (Scanco Medical, Brüttisellen, Switzerland) system. Here, 10-µm voxel size, 55 KVp, 0.36 degrees rotation step (180 degrees angular range), and a 1,000-ms exposure per view were used for the scans that were performed in 70% alcohol. The Scanco µCT software (HP, DECwindows Motif 1.6) was used for three-dimensional (3D) reconstruction and viewing of images. After 3D reconstruction, volumes were segmented using a global threshold of 400 mg HA/mm³. Bone volume (BV), total volume (TV), directly measured bone volume fraction (BV/TV), connectivity density (Conn-Dens.), trabecular number (Tb.N), trabecular thickness (Tb.Th), trabecular separation (Tb.Sp), total (in this case, bone) mineral density (TMD), and surface-to-volume ratio (BS/BV) were calculated.

### Biomechanical testing

Torsional test to failure was performed on 28-day specimens. The proximal and distal ends of the bone were then potted in brass cylinders. Tibiae were subjected to torsion at 1°/s. The gauge length was measured for each specimen. Torque and displacement data were recorded at a rate of 20 Hz. Maximum torque, torsional rigidity, twist to failure and twist to max load (units: radians normalized by gauge length), and toughness were calculated using a custom routine in LABVIEW. Torsional rigidity was defined as the slope of the initial portion of the torque-twist curve, where twist was normalized by gauge length and toughness was defined as the area beneath the curve until the maximum torque was reached.

### Statistics

All analyses were performed by the author with Prism 8 or 9 (GraphPad Software, La Jolla, CA, USA). Means and standard deviation (SD) were calculated for all data sets. For glucose tolerance testing, geometric AUC was calculated for each group to compare glucose tolerance. Body weight, glucose tolerance testing, blood glucose testing, and histological lesions of the liver were calculated by a two-tailed unpaired *t*-test analysis to assess the differences in lean and HFHS groups. CFU was calculated by a two-tailed unpaired *t*-test analysis to assess the differences between the FX and MRSA groups in lean and HFHS groups. Fracture healing parameters (RUST score) were calculated by one-way ANOVA with Tukey’s *post-hoc* analysis to assess the differences among groups. μCT and torsional strength were calculated by a two-tailed unpaired *t*-test analysis to assess the differences among groups. All analyzed significance results were directly indicated in the figures. A *post-hoc* power analysis was performed using sterile fracture data among wild-type mice for biomechanical testing, with a power of 80% and type I error 5%. The current study was powered to identify 12.2 N/mm difference in stiffness, 246 N mm^2^/rad difference in torsional rigidity, and 11 Nmm max torque%.

## Results

### Maintenance of a high-fat and high-sucrose diet contributed to the development of T2DM phenotypes in mice

**Figure 1:**
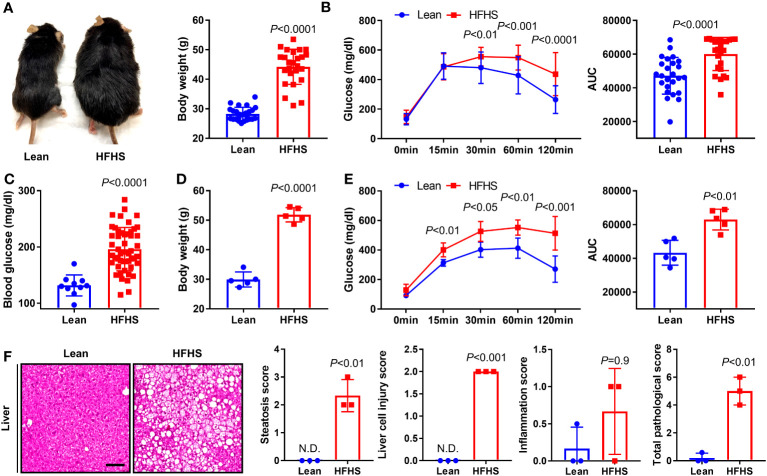
To construct an animal model of diabetes, C57BL/6J mice were maintained on an high-fat and high-sugar (HFHS) diet for 3 and 6 months; lean-fed mice were used as a control group. **(A)** To verify the development of obesity, we measured morphological changes and body weight in lean and HFHS diet models at 3 months (*n* = 25 per group). **(B)** Glucose tolerance test (GTT) results were monitored for 120 min, and the area under the curve (AUC) at 3 months was analyzed (*n* = 25 per group). **(C, D)** Blood glucose levels and body weight in lean and HFHS models at 6 months were measured (*n* = 5–50 per group). **(E)** GTT results were monitored for 120 min, and the AUC at 6 months was analyzed (*n* = 5 per group). **(F)** Histological lesions of the liver in lean and HFHS models at 6 months were measured using total pathological score (i.e., steatosis, liver cell injury, and inflammation scores) (*n* = 3 per group). Error bars show means ± SD. Two-tailed unpaired t-test analysis was used to assess statistical significance when compared to the lean group. N.D., not detected.

At 3 months after initiation of an HFHS diet, physiologic obesity began to be obvious in the HFHS mice. The average body weight of mice in the lean group was 28 g compared to an average body weight of 45 g in mice fed an HFHS diet, an increase of approximately 60% over the lean group (**Figure 1A**). GTT results were used to screen for T2DM and demonstrated significantly increased glucose tolerance in the HFHS group, resulting in an increase in the AUC relative to that in the lean group (**Figure 1B**). Blood glucose levels, average body weight, and glucose tolerance in the HFHS group were significantly greater than those of the lean group. These differences persisted over a 6-month period (**Figures 1C–E**). T2DM is one of the important risk factors for the faster progression of nonalcoholic fatty liver disease, fibrosis, and cirrhosis, which presents steatosis, injury, and inflammation (Targher et al., 2021). Our T2DM model showed that on the total histopathological hepatic score with respect to steatosis, liver cell injury, and inflammation score, an increased number of liver lesions were observed in the HFHS group compared to the lean group (**Figure 1F**). Therefore, we confirmed the presence of various lesions in the T2DM model and used this to assess the correlation between T2DM with tibial open fracture healing and osteomyelitis.

### Evaluation of tibial open fracture healing and osteomyelitis in the setting of T2DM

**Figure 2:**
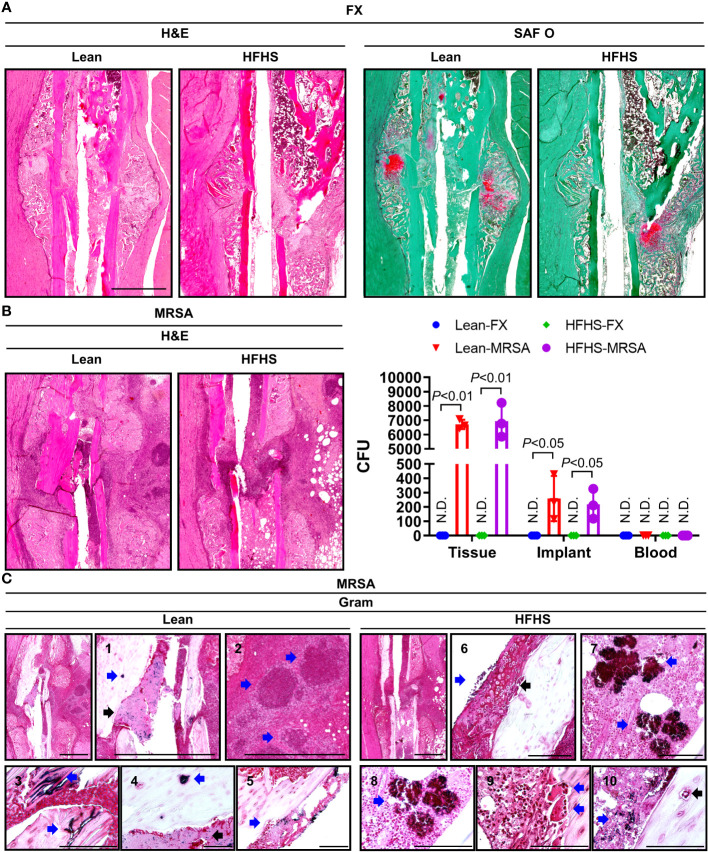
Histological assessment of open tibial fracture and MRSA-induced osteomyelitis. **(A–C)** MRSA (1 × 10^6^ CFU)-infected tibiae observed 2 weeks after open fracture induction in a lean- and HFHS-fed model for 6 months. **(A)** Paraffin-embedded tissues were stained with H&E and SAF O; the pink area stained with SAF O suggested chondrogenic differentiation. **(B)** Paraffin-embedded tissues were Gram-stained, and the number of colony-forming units (CFUs) in tissue, implants, and blood was measured (*n* = 3 per group). **(C)** Paraffin-embedded tissues were Gram-stained; C1–C5 were derived from lean-fed models and C6–C10 were derived from HFHS-fed models. C1 and 4: The blue arrow indicates MRSA-infected osteocyte lacunae, and the black arrow indicates the presence of MRSA in areas of fibrinoid necrosis. C2: Blue arrows indicated the formation of abscess communities. C3: The blue arrow indicates MRSA colonization within necrotic bone. C5: The blue arrow indicates the presence of MRSA-infected cells in the bone marrow space. C6: The blue arrow indicates the presence of MRSA-infected chondrogenic cells, and the black arrow highlights chondrogenesis. C7 and C8: Blue arrow is indicative of MRSA burden. C9: The blue arrow points to MRSA-infected osteoclasts in sites of bone resorption. C10: The blue arrow indicates MRSA-infected osteocyte lacunae, and the black arrow indicates MRSA-infected areas of fibrinoid necrosis. Error bars show means ± SD. Two-tailed unpaired *t*-test analysis was used to assess statistical significance when compared to the lean-FX group or HFHS-FX group. N.D., not detected.

Fracture healing was evaluated histopathologically at the 2-week time point in lean and HFHS-fed C57BL/6 male mice. Robust fracture callus and chondrogenesis were observed in the lean group, but callus formation and chondrogenesis were inferior in the HFHS group (**Figure 2A**). MRSA resulted in osteomyelitic stigmata such as severe inflammation, bone resorption, necrotic bone, sequestrum, involucrum, and fibrinoid necrosis in both the lean and HFHS groups (**Figure 2B**). A high MRSA bioburden—as evidenced by a large number of MRSA CFU—was observed at the fracture site and on the harvested implant, which did not differ between the lean and HFHS groups. MRSA was not detected in the bloodstream, suggesting that bacteremia did not occur. Gram staining revealed the presence of MRSA (**Figure 2C**). MRSA was also present within osteocyte lacunae, and widespread colonization was observed within necrotic bone. Interestingly, we identified that a small number of intracellular MRSA were present in osteoclasts at the site of bone resorption.

### Dual antibiotic treatment subsides infection but impaired fracture healing persists in T2DM mice

**Figure 3:**
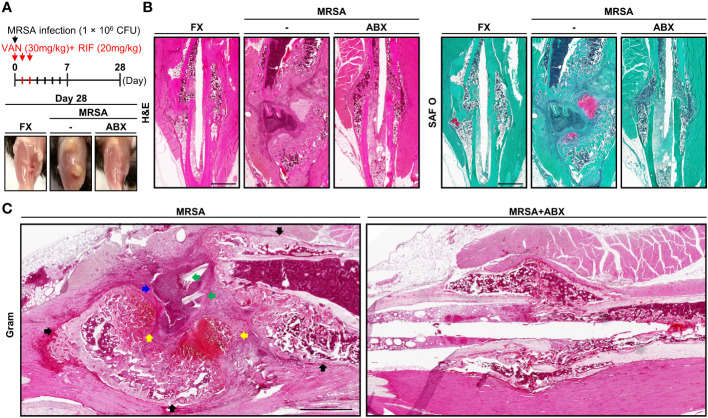
Histological assessment of MRSA-induced osteomyelitis with antibiotic treatment. (**A**) MRSA-infected (1 × 10^6^ CFU) tibiae in mice fed an HFHS diet for 6 months, then treated with vancomycin (30 mg/kg) and rifampin (20 mg/kg) for 3 days (*n* = 3 per group). After 4 weeks, physiological changes characteristic of T2DM were observed, and representative images were generated. (**B, C**) Paraffin-embedded tissues were stained with H&E and SAF O. (**C**) The blue arrow indicates the formation of abscess communities. Black arrows point to involucrum formation. Green arrows indicated MRSA colonization within the necrotic bone. Yellow arrows are suggestive of chondrogenic differentiation.

We designed and evaluated a systemic dual antibiotic treatment comprising vancomycin and rifampin, demonstrating that dual antibiotic treatment reduced osteomyelitis lesions (**Figure 3A**). Four weeks postoperatively, the HFHS group demonstrated near-complete unicortical union on one side of the fracture, while the other side progressed to nonunion (**Figure 3B**). As in the 2-week model, a large number of adipocytes were observed in the callus of HFHS mice. At 4 weeks, HFHS mice with MRSA infection demonstrated more severe inflammation, bone resorption, necrotic bone, sequestrum and involucrum formation, and fibrinoid necrosis than did 2-week models (**Figures 3B, C** **)**. Following treatment with combinatorial antibiotics, MRSA was not visualized on histopathological analysis, but impaired fracture healing persisted relative to the noninfected group. These findings suggest that systemic antibiotic treatment alone was not sufficient to accomplish fracture healing in T2DM mice.

### Adjuvant iPTH treatment improves fracture healing in T2DM mice

**Figure 4:**
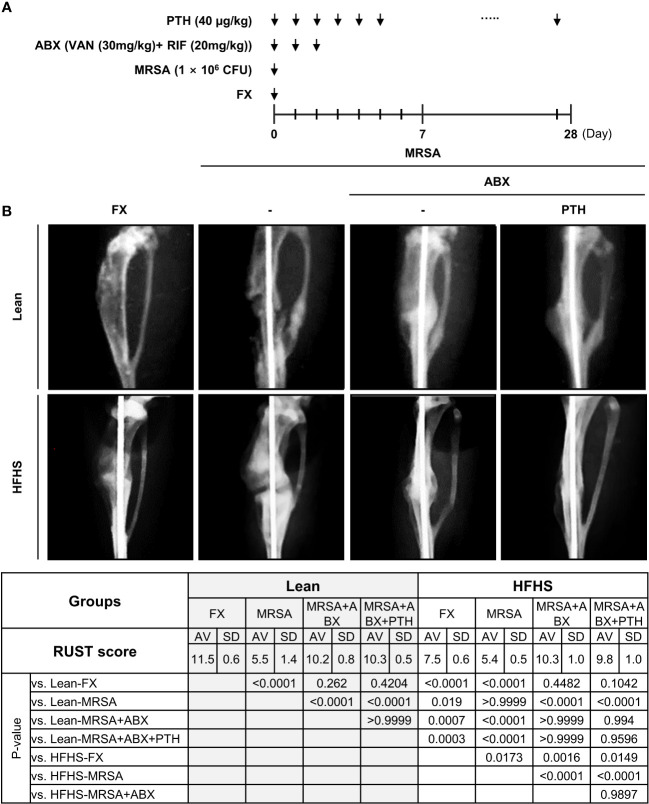
**(A)** Experimental design and radiographic assessment of MRSA-infected fracture healing. A surgical open fracture model was performed with MRSA infection (1 × 10^6^ CFU) on day 0 in lean- and HFHS-fed models. Antibiotic groups were treated with vancomycin (30 mg/kg) and rifampin (20 mg/kg) for 3 days (*n* = 4–6 per group). In the PTH condition, PTH was administered for 27 days. **(B)** Mice were sacrificed on day 28. Fracture union was measured radiographically using the radiographic union score for tibial fractures (RUST) according to the guidelines. Error bars show means ± SD. One-way ANOVA with Tukey’s *post-hoc* analysis was used to assess statistical significance.

We designed a systemic dual antibiotic combination with adjuvant iPTH treatment, which evaluated fracture healing by RUST score analysis (**Figure 4A**). Lower RUST scores were observed in the HFHS group relative to those of the lean mouse group, suggesting that fracture healing was delayed in our HFHS models independent of infectious status (**Figure 4B**). Reduced RUST scores were also observed in both MRSA-infected lean and HFHS mice compared to the noninfected group, which partially improved with antibiotic treatment. Interpretation of plain radiographs did not demonstrate higher increments of RUST scores between the antibiotics alone group vs. combined antibiotics + adjuvant iPTH treatment.

**Figure 5:**
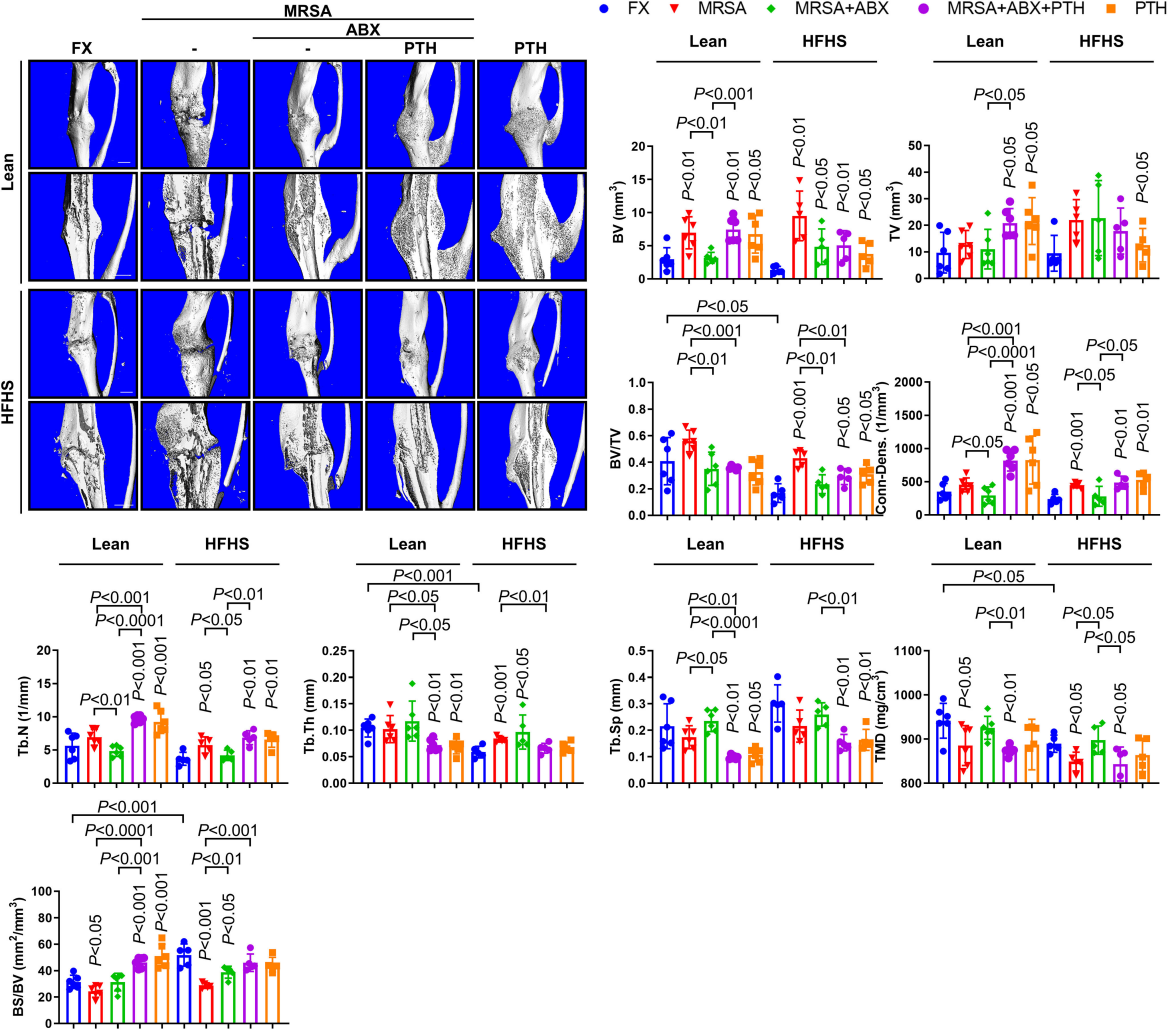
Micro-computed tomography (μCT) assessment of MRSA-induced osteomyelitis following antibiotics and PTH treatment. MRSA-infected (1 × 10^6^ CFU) tibiae in lean- and HFHS-fed models were treated with vancomycin (30 mg/kg) and rifampin (20 mg/kg) or 3 days (*n* = 5–6 per group). Antibiotic and PTH treatments were administered in the experimental models, while PTH treatment alone was used in the positive control. Tibial tissues were measured for callus formation and union changes using μCT analysis with respect to bone volume (BV), total volume (TV), BV/TV, connectivity density (conn-dens), trabecular number (Tb.N), trabecular thickness (Tb.Th), trabecular separation (Tb.Sp), total mineral density (TMD), and bone surface/bone volume (BS/BV). Error bars show means ± SD. Connectivity density and trabecular number increased with iPTH treatment in MRSA-infected T2DM mice. Two-tailed unpaired *t*-test analysis was used to assess statistical significance when compared to the lean-FX group and/or HFHS-FX group.

The quality of callus formation and bone microarchitecture were analyzed via μCT analysis. Bone volume/total volume (BV/TV), Tb.Th, TMD, and bone surface/bone volume (BS/BV) were lower in the HFHS group compared with those in the lean group (**Figure 5**). Lean mice almost consistently demonstrated union at the fracture site, while delayed fracture healing and nonunion were observed in the HFHS mice. The μCT parameters were improved with iPTH treatment. Fragmented necrotic bone and involucrum formation were observed in MRSA-infected fractures. Antibiotic treatments reduced necrotic bone and involucrum, although fracture healing remained impaired compared to that of the noninfected controls. Adjuvant PTH treatment with antibiotic administration increased callus formation and the rate of fracture union in both the lean and HFHS groups. Overall, these results suggest that iPTH treatment delivered adjuvant to antibiotics improves callus formation and strength-conferring parameters of bony microarchitecture in the setting of MRSA-infected fractures in T2DM mice that show reduced fracture healing potential even without MRSA infection.

### Biomechanical parameters of fracture healing

**Figure 6:**
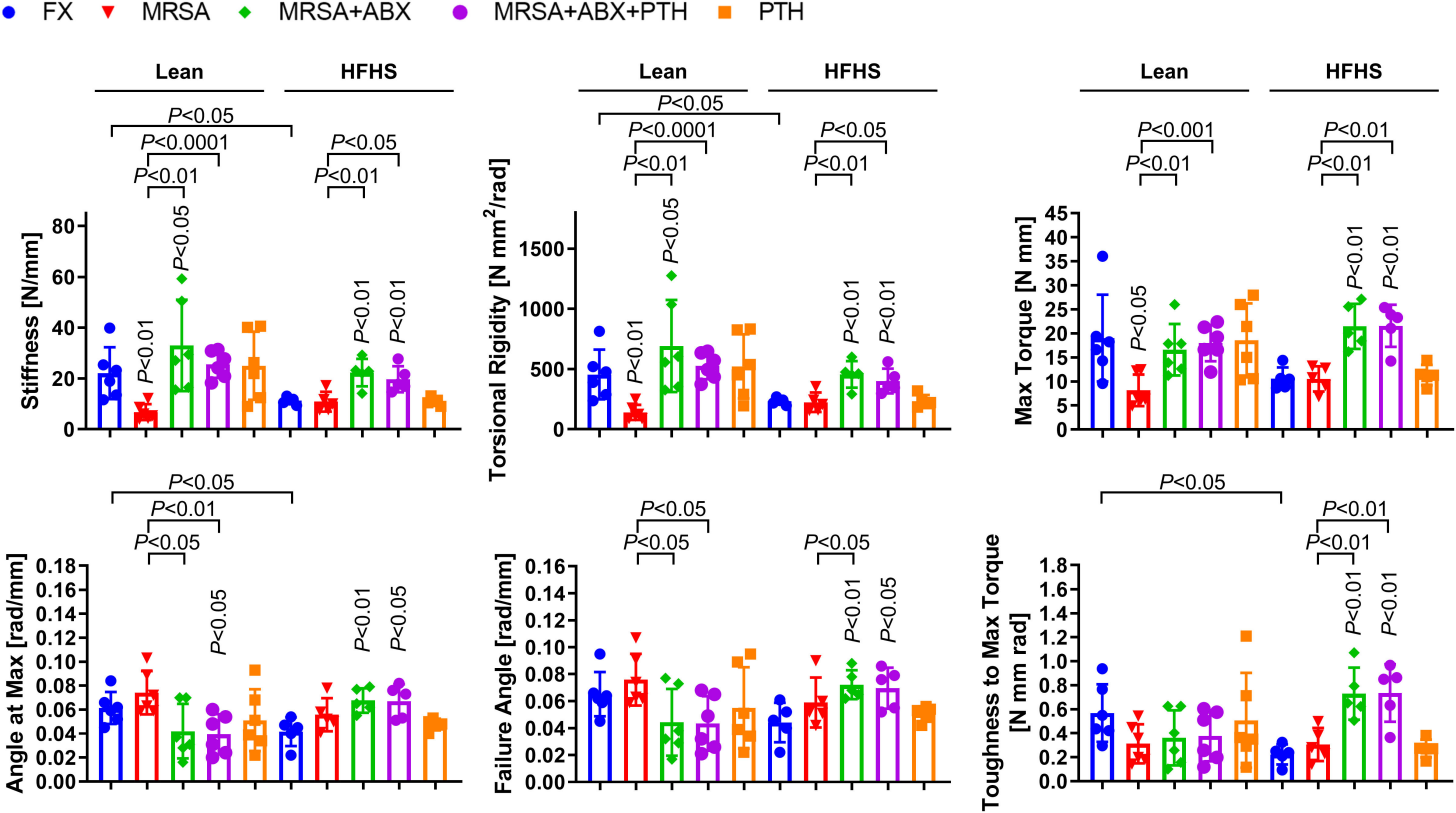
Mechanical strength assessment of MRSA-infected osteomyelitic bone in lean-fed and HFHS-fed mice. MRSA-infected (1 × 10^6^ CFU) tibiae in lean- and HFHS-fed models were treated with vancomycin (30 mg/kg) and rifampin (20 mg/kg) for 3 days (*n* = 5–6 per group). Antibiotic and PTH treatments were administered in the experimental models, while PTH treatment alone was used in the positive control. Tibial tissues were measured with respect to mechanical strength parameters such as bone stiffness, torsional rigidity, max torque, angle at max, failure angle, and toughness to max torque. Error bars show means ± SD. Two-tailed unpaired *t*-test analysis was used to assess statistical significance when compared to the lean-FX group and/or HFHS-FX group.

Biomechanical testing results showed that fracture healing was inferior and bone quality was poor in the HFHS group compared to those in the lean group. Torsional testing of fracture tibiae at 4 weeks revealed significantly reduced bone stiffness, torsional rigidity, angle at max, and toughness to maximum torque in the HFHS group compared to the lean group (**Figure 6**). In the lean group, MRSA-infected fractures showed significantly reduced bone stiffness, torsional rigidity, and max torque compared to the noninfected group. Conversely, no significant differences were observed in biomechanical strength parameters between the infection and non-infection groups in HFHS-fed mice, both of which parameters were lower than those of the lean noninfected mice, suggesting profoundly inferior fracture healing in T2DM mice without infection compared to lean mice. Importantly, antibiotic treatment in the lean- and HFHS-fed groups resulted in increases in most biomechanical strength parameters, including bone stiffness, torsional rigidity, and max torque. Most of these biomechanical parameters in HFHS-fed mice were restored to levels comparable to those in lean mice, suggesting potential for enhancement of fracture healing with iPTH even in T2DM with infection at the fracture site.

## Discussion

We explored the beneficial effects of iPTH in T2DM infected fractures, as there is no literature on this issue. Teriparatide administration yielded no significant benefit with respect to RUST scores that are judged by plain radiographs. However, the bony microstructure of the callus such as connectivity density and trabecular number on μCT improved with iPTH in both lean- and HFHS-fed mice. Lean-fed mice demonstrated more significant improvement with iPTH in callus microstructure compared to HFHS mice under both infected and sterile conditions. This means that the anabolic effects of iPTH in T2DM mice are inferior to those of lean mice, but the potential for iPTH-induced anabolic effects is not abolished in MRSA-infected T2DM bone. The discrepancy in RUST scores and μCT parameters could be due to lower image resolution of digital radiography for small mouse tibiae in comparison to μCT.

We admit that our assessment was done for fracture healing after intramedullary nailing. Fracture healing is different with respect to stiffness of fracture fixation and fracture gap. Primary interosseous healing is dependent upon direct bone fragment contact with plate and screw fixation with a gap of less than a millimeter, high stability, and minimal strain. Secondary endochondral healing predominates after treatment with cast immobilization or intramedullary nailing and proceeds via an initial inflammatory phase, followed by soft callus formation, hard callus formation, and fracture remodeling (Giannoudis et al., 2007; Giannoudis et al., 2011). Although patients with T2DM remain at increased risk of insufficiency fracture, they sometimes possess normal or slightly increased bone density, although this bone is suspected to be of lower quality relative to normal bone (Bouillon, 1991; Dhaliwal et al., 2014; Ma et al., 2017; Marin et al., 2018). Diabetic bone disease is characterized by reduced bone turnover and structurally inferior, biomechanically weaker bone, thus culminating in increased fracture risk (Blakytny et al., 2011; Sellmeyer et al., 2016; Napoli et al., 2017). The homeostatic balance between osteoblasts and osteoclasts within bone is disrupted in the setting of DM, resulting in impaired osteoblastic differentiation from mesenchymal precursors and increased adipogenesis within bone marrow, resulting in microarchitectural disruption (Blakytny et al., 2011). These altered biological properties in T2DM mice may predispose to more aggravation of fracture healing by infection and less anabolic responses to iPTH. We observed a reduction in fracture callus formation and bone quality in HFHS-fed mice compared to lean mice. iPTH administration increased callus formation in both lean and HFHS groups, suggesting remaining anabolic responses to iPTH in T2DM mice. iPTH increased the rate of bony union in T2DM mice, but μCT parameters of bony architecture remained lower compared to their lean counterparts. Several studies showed that metabolic perturbations in the setting of DM result from the nonenzymatic glycosylation of advanced glycation end products (AGEs) within bone, which promotes the cross-linking of type I collagen fibrils and disruption of the bone extracellular matrix, thereby increasing the stiffness and fragility of bone and reducing its biomechanical strength (Blakytny et al., 2011; Zhou and Xiong, 2011; Saito et al., 2014). In the present investigation, iPTH did not significantly improve the biomechanical strength of the infected treated fractures compared to the antibiotic-treated condition alone in both lean and HFHS-fed mice, while bone microarchitecture improved with adjuvant iPTH. This could be due to biomechanical testing conditions that were geared for normal mouse long bones. As fracture healing is inferior and delayed in T2DM mice, T2DM mice may require more time to show anabolic effects of iPTH.

DM is a well-recognized and well-characterized pro-inflammatory condition secondary to excessive activation of the receptor for advanced glycation end products (RAGE) immunoglobulin superfamily by RAGE ligands produced under conditions of hyperglycemia and oxidative stress (Zhou and Xiong, 2011; Kierdorf and Fritz, 2013). The early transient inflammatory phase of fracture healing is critical, as blood vessel disruption results in hematoma formation, innate immune cell recruitment and trapping, and chemokine release (Giannoudis et al., 2007). Secreted growth factors and cytokines include tumor necrosis factor-alpha (TNF-α), interleukin-1 beta (IL-1β), interleukin-6 (IL-6), and macrophage chemoattractant protein-1 (MCP-1), which promote mesenchymal stem cell recruitment to the fracture site (Giannoudis et al., 2007). Improper regulation of inflammation at the fracture site—with respect to inflammatory response magnitude, duration, and sequential timing—is associated with impaired callus formation and fracture healing (Carlsten, 2005; Loi et al., 2016). Inflammatory markers upregulated in DM also reduce angiogenesis and endochondral bone formation. The excessive systemic inflammation characteristic of DM and diabetic bone disease results in prolonged inflammation at the fracture site, which disrupts the initial physiologic inflammatory response necessary for angiogenesis, regulated immune cell recruitment, and soft callus formation (Bouillon, 1991; Blakytny et al., 2011; Jiao et al., 2015; Henderson et al., 2019; Yu et al., 2020b). Similar to the prolonged pro-inflammatory state induced by DM, infection induces the release of cytokines and chemokines, induces osteoclastogenesis, and suppresses osteoblast differentiation from mesenchymal stem cell precursors (Lew and Waldvogel, 1997; Jorge et al., 2010). Our previous study showed that iPTH enhanced murine fracture healing in HFHS diet-induced T2DM, suggesting potential anabolic booster for infection-impaired fracture healing in T2DM even in the presence of T2DM (Alder et al., 2020b).

Bacterial contamination has been demonstrated to disrupt the process of callus formation. Histologic studies suggest that this impedance to callus formation may be attributed to persistent neutrophil infiltration both locally within infected fracture sites and elevated systemic neutrophil levels (Lovati et al., 2016). This sustained inflammatory activity dysregulates osteogenic differentiation, the production of progenitor cells from the bone matrix, and the recruitment of monocytes and macrophages to the site of infected fracture site, thus hindering both angiogenesis and osteogenesis necessary for fracture healing (Bastian et al., 2011; Schmidt-Bleek et al., 2015; Kovtun et al., 2016; Bastian et al., 2018). Previous studies by authors and other investigators described that vancomycin, which comprises the first-line therapy for osteomyelitis caused by MRSA infection, does not fully eradicate infection due to its inability to penetrate the intracellular space (Alder et al., 2020a; Yu et al., 2020a; Cahill et al., 2021). Rifampin, which can achieve intracellular penetrance, is effective against intracellular MRSA and MRSA-induced osteomyelitis and septic arthritis (Alder et al., 2020a; Cahill et al., 2021; Kwon et al., 2021). Histologic analysis demonstrated the presence of MRSA within osteocyte lacunae and osteoclasts at infected fracture sites, in addition to the presence of bone resorption, fibrinoid necrosis, sequestrum, and involucrum formation. While combined vancomycin and rifampin therapy more effectively resolved infection in our murine models of infected tibial shaft fracture, it did not result in the resolution of fracture healing in either lean or diabetic mice.

On a cellular level, DM upregulates tissue adipogenesis, including within bone, such that mesenchymal stem cell bone progenitors increasingly differentiate into adipocytes rather than osteocytes that beget osteoblast proliferation (Sharma et al., 2011; Brown et al., 2014). Increased adipose tissue deposition—as observed in our HFHS diabetic, but not lean wild-type, mice—at the fracture site impedes fracture callus formation and local healing. Counter to expectations, in our models of infected diabetic fracture healing, more robust bone formation was achieved in HFHS mice in the setting of MRSA infection following antibiotic therapy than in the sterile fracture condition. We postulate that killed bacteria stimulate an inflammatory response that may be pathologic, similar to involucrum formation in the setting of osteomyelitis and hypertrophic fracture nonunion due to excessive inflammation in the setting of DM and infection. This is consistent with previous observations in diabetic mice demonstrating increased periosteal callus formation in the setting of *S. aureus* peri-implant infection (Farnsworth et al., 2018).

It should be noted that the HFHS diet-induced mouse model that was used in this study resulted in less dramatic metabolic abnormalities compared to other T2DM models, such as genetic strains or chemically induced models. Follow-up studies are needed to investigate the use of teriparatide in a chronic infection model, as well as possible disparities in the benefits of adjunctive teriparatide treatment following conventional antibiotic therapy in small animal models of poor versus well-managed glycemic control.

There are some limitations to our results. Surprisingly, the MRSA+ABX group scored higher in radiographic union, although the bone volume is decreased. We speculate that a higher initial inflammatory response stimulated by a bacterial load may contribute to this finding or a direct stimulatory effect of rifampin. It is noted that BV is decreased, raising the possibility that bone quality is decreased in this case. However, mechanistic investigation into this effect was beyond the primary goal of this study and would be a possible avenue for investigation in follow-up studies. Adjunctive iPTH treatment did not significantly improve the biomechanical strength of infected antibiotic-treated tibiae over the antibiotic-alone condition, although several facets of bone microarchitecture improved with teriparatide treatment compared to the systemic antibiotic-treated condition. PTH treatment did not significantly improve the biomechanical strength compared to antibiotic treatment alone in lean- and HFHS-fed groups. PTH treatment under noninfectious conditions also did not yield significant changes in biomechanical strength. This may suggest that longer time points are needed to see iPTH effects on biomechanical healing when the fracture healing process is undermined by T2DM and infection. In this study, 28 days was chosen, as this is a commonly used end point for biomechanical analysis in murine long bone fracture healing studies. Another limitation is the modest number of mice included in the biomechanical testing groups, which possibly contributes to type II error in this study. It should be noted that torsional biomechanical testing was used primarily in this analysis, as a nondestructive 3-point bend test was determined to be not ideal for assessing mechanical differences in the current model. The single loading point focuses all of the force over a small region, which is problematic, as the callus area is relatively large. Additionally, the callus is made of thin immature bony tissues, increasing the likelihood of local deformation during testing. For these reasons, it was decided that torsional testing is the most accurate gauge of biomechanical strength. The definition of fracture healing remains to be settled. RUST scores based on plain radiographs are a semiquantitative scoring system on relatively low-resolution radiographs, posing issues of ceiling effects. Another area that can be of further investigation is comparing fracture healing in mice undergoing intramedullary nailing versus rigid fixation with plate and screws, representing secondary endochondral bone repair and primary bone healing. Increased trabecular connectivity and numbers with combinatorial antibiotics and iPTH treatment on μCT could be related to decreased adiposity of bone marrow in HFHS diet-induced T2DM mice. Lastly, the current study only includes structural biomechanical data for primary analysis. Micromechanical analysis could be accomplished with nano-indentation testing. Although this is germane to clinical treatment results, this study is limited in that biological and cellular data are limited, which may be addressed by future investigations.

We created a murine model of infected T2DM tibial shaft fracture and characterized changes in fracture healing with systemic antibiotic therapy alone and combined systemic antibiotic and iPTH treatment. Adjuvant iPTH treatment may be considered to enhance impaired fracture healing in patients with T2DM that is further complicated with infection.

## Data availability statement

The original contributions presented in the study are included in the article/supplementary material. Further inquiries can be directed to the corresponding authors.

## Ethics statement

The animal study was approved by Yale University Institutional Animal Care and Use Committee (IACUC; Number: 2020-20129). The study was conducted in accordance with the local legislation and institutional requirements.

## Author contributions

H-KK, SC, and KY designed and performed experiments and analyzed the data. FL and H-KK conceived and designed the overall study as the principal investigator. CD, KA, and JB performed the experiments. JJ performed the experiments on hepatic steatosis. All authors contributed to the writing and editing of the manuscript. All authors contributed to the article and approved the submitted version.
